# Copper Nanowires Modified with Graphene Oxide Nanosheets for Simultaneous Voltammetric Determination of Ascorbic Acid, Dopamine and Acetaminophen

**DOI:** 10.3390/molecules24122320

**Published:** 2019-06-24

**Authors:** Wanting Hao, Yuchan Zhang, Jingchuan Fan, Handeng Liu, Qi Shi, Weichi Liu, Qianyu Peng, Guangchao Zang

**Affiliations:** 1 Institute of Life Science, and Laboratory of Tissue and Cell Biology, Lab Teaching & Management Center, Chongqing Medical University, Chongqing 400016, China; HWThally@163.com (W.H.); zhangyc@cqmu.edu.cn (Y.Z.); fanjc@cqmu.edu.cn (J.F.); hdliu@cqmu.edu.cn (H.L.); sq1139188112@163.com (Q.S.); lwc805447132@163.com (W.L.); 13996689178@163.com (Q.P.); 2 Key Laboratory of Laboratory Medical Diagnostics of Education, Department of Laboratory Medicine, Chongqing Medical University, Chongqing 400016, China

**Keywords:** cyclic voltammetry, differential pulse voltammetry, synergistic effect, antioxidant, excitatory neurotransmitter, analgesic

## Abstract

Copper nanowires (Cu NWs) were modified with graphene oxide (GO) nanosheets to obtain a sensor for simultaneous voltammetric determination of ascorbic acid (AA), dopamine (DA) and acetaminophen (AC). The nanocomposite was obtained via sonication, and its structures were characterized by scanning electron microscopy (SEM), X-ray diffraction (XRD) and energy-dispersive X-ray spectroscopy (EDS). The electrochemical oxidation activity of the materials (placed on a glassy carbon electrode) was studied by cyclic voltammetry and differential pulse voltammetry. Due to the synergistic effect of Cu NWs and GO, the specific surface, electrochemical oxidation performance and conductivity are improved when compared to each individual component. The peaks for AA (−0.08 V), DA (+0.16 V), and AC (+0.38 V) are well separated. The sensor has wide linear ranges which are from 1–60 μM, 1–100 μM, and 1–100 μM for AA, DA, and AC, respectively, when operated in the differential pulse voltammetric mode. The detection limits are 50, 410 and 40 nM, respectively. Potential interferences by uric acid (20 μM), glucose (10 mM), NaCl (1 mM), and KCl (1 mM) were tested for AA (1 μΜ), DA (1 μΜ), and AC (1 μΜ) and were found to be insignificant. The method was successfully applied to the quantification of AA, DA, and AC in spiked serum samples.

## 1. Introduction

Ascorbic acid (AA), dopamine (DA), and acetaminophen (AC) are considered to be crucial molecules that commonly coexist in human metabolic fluid [1,2,3,4]. Ascorbic acid, a vital vitamin in the human diet, has a major role in bioelectrochemistry, neurochemistry and clinical diagnostic applications [5]. Such antioxidants help prevent acquired immune deficiency syndrome (AIDS), cancer, scurvy, hepatic disease, mental illness, and the common cold [6,7,8,9,10]. Dopamine, a chemical compound found in the brain, acts as a significant excitatory neurotransmitter to regulate system functions, such as renal, hormonal systems, and the central nervous system [1,11,12,13]. A lack of DA will lead to nervous system disease, for instance schizophrenia or Parkinson’s disease [2,14]. Acetaminophen, a well-established over the counter (OTC) drug used worldwide, is well known as an analgesic and antipyretic for relieving pain associated with toothache, headache, muscle aches, colds, fevers, postoperative pain, etc. [15,16,17]. Moreover, it has been reported as a potential prodrug for resistant cancer therapy [18]. Overdoses of AC can induce side effects, such as severe and sometimes fatal hepatotoxicity and nephrotoxicity [1,19,20]. Higher concentration levels of AA, DA and AC are associated with a higher risk of various diseases linked to human health management and treatment. Thus, exploring a high-performance analytical method to realize good sensitivity to these three compounds simultaneous determination is an urgent need.

Electrochemical techniques are well known, and fast, simple, sensitive, selective, reliable and low-cost characteristics have been developed alongside analysis of multiple components in biological matrices. Lots of studies have reported the feasibility of realizing the simultaneous determination of two to five components commonly containing uric acid (UA), DA, and AA. Nevertheless, it is very difficult to simultaneously test AA, DA, and AC, as the three chemical compounds have semblable electrochemical performances, and their oxidation peak potentials are not clearly separated on many electrodes, greatly complicating their electrochemical discrimination [16,21,22]. Only a minority of research efforts have been developed with various electrodes for simultaneous determination of AA, DA, and AC. For example, glassy carbon electrodes were modified with Au/ZnO/N-doped graphene hybrid nanostructure [1] and phenylethynyl ferrocene thiolate-modified Fe_3_O_4_@Au nanoparticles (NPs) were coupled with a graphene sheet/chitosan [16], individually, and a carbon paste electrode was modified with a PbS nanoparticles Schiff base [22]. These have been reported, and they demonstrate the electrochemical activity which occurs simultaneously with the three compounds oxidation. Although exhibiting improved electrochemical oxidization for peak separations of the three compounds, these electrodes are still not appropriate mass production objects for fabricating corresponding electrochemical sensors due to their high price and complexities resulting from many kinds of materials.

However, the aforementioned graphene nanosheet and its derivatives, such as N-doped graphene [1], as well as reduced graphene oxide (rGO) [23] and graphene oxide (GO) [24] based nanocomposites, display great potential application for fabricating electrochemical sensors, with superior performance attributable to good chemical stability, large specific surface area, admirable conductivity, relatively easy chemical modification, strong mechanical strength and flexibility, and high electrochemical activity for biomolecules. Among the various graphene derivatives, it is worth noting that GO is rich in epoxy, phenol, hydroxyl, and carboxylic acid groups, which is concomitant with strongly hydrophilic endowed GO good performance to modulate the electrode surface structure and properties [25,26,27]. For instance, multi-walled carbon nanotubes/GO/gold nanorods/glassy carbon electrode (GCE) shows an ultra-sensitive detection of AA with a low detection limit (LOD) of 0.85 nM [24]. In addition, a Co(II)-based zeolitic imidazolate framework (ZIF-67) and GO nanocomposite modified GCE was successfully simultaneously tested for DA and UA [28], and an electrochemical sensor based on GO-1,4-xylenediamine-Mn_2_O_3_ nanospheres nanocomposite was stable and sensitive for simultaneous determination of AA and paracetamol [29]. Furthermore, AgNPs-GO-poly(L-arginine) composite-modified GCE shows simultaneous determination of AA, DA, UA, and L-tryptophan [30]. The poly(glycine)/GO composite-based sensor significantly amplifies the electrochemical signal for the simultaneous detection of UA, DA, guanine and adenine [31]. These studies reveal that GO-based sensors have been proved to be a favourable alternative for application in simultaneous detection.

Metal nanowires, especially copper nanowires (Cu NWs), with outstanding electrochemical performance, such as efficient electron transfer and high catalytic activity, are promising materials for the development of electrochemical sensors [32]. Thus, in this study, the specific property of Cu NWs and GO were combined to prepare a nanocomposite (Cu NWs–GO) with synergistic integration of individual component to enhance the electrochemical characteristics for AA, DA, and AC simultaneous determination. This hybrid nanocomposite was prepared by a general protocol of ultrasound mixing. Differential pulse voltammetry (DPV) was carried out to investigate the electrochemical properties, which exhibit a wide linear range with a low detection limit of AA, DA, and AC. In addition, the proposed assay also demonstrates good selectivity against other conventional interfering substances. It also evaluates the reported method upon application in human serum samples, indicating the potential applicability for simultaneous determination of these analytes.

## 2. Results and Discussion

### 2.1. Characterization of the Copper Nanowires–Graphene Oxide (Cu NWs–GO) Nanocomposite

**Figure 1:**
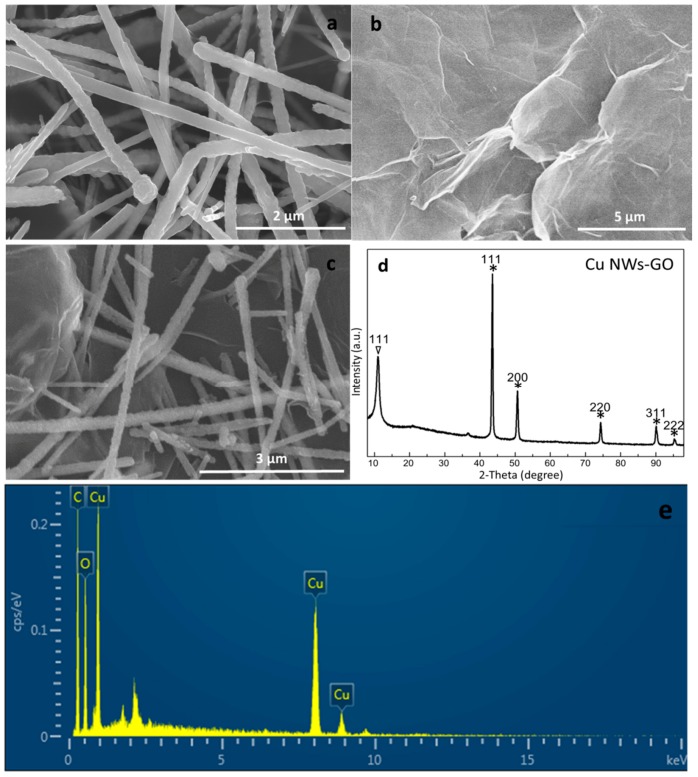
The scanning electron microscope (SEM) images of (**a**) copper nanowires (Cu NWs), (**b**) graphene oxide (GO) and (**c**) Cu NWs–GO nanocomposite. (**d**) The X-ray diffraction (XRD) patterns of Cu NWs–GO nanocomposite. The asterisks represent the 2θ values of Cu NWs surface. (**e**) The energy-dispersive X-ray (EDS) spectrum of Cu NWs–GO nanocomposite.

The typical scanning electron microscope (SEM) images of synthesized nanomaterials including Cu NWs, GO, and Cu NWs–GO nanocomposite are displayed in Figure 1a–c. One can see in Figure 1a that the size of Cu NWs is well-proportioned with a diameter of 150 nm ± 50 nm—the same as that reported in literature [33], endowing it with good sensing performance. The morphology of GO is crumpled (Figure 1b), giving it good flexibility and high specific surface area for the immobilization of a great many substances. The SEM image of the nanocomposite (Figure 1c) notably shows that Cu NWs are well distributed and interconnect on the GO surface, implying that the high specific surface with loose structure enhances catalytic activity. The successful formation of Cu NWs–GO nanocomposite is further demonstrated by X-ray diffraction (XRD), as shown in Figure 1d. The XRD results of the Cu NWs–GO nanocomposite reveal intense and sharp peaks centered at 2θ values of *10.834°, 43.297°, 50.433°, 74.130°, 89.931°, and 95.139°, perfectly matching the *(111), (111), (200), (220), (311), and (222) Miller indices, respectively. The 2θ value and Miller Index preceded by the asterisk is similar to the GO surface (Figure S1a) [34,35,36], and the other peaks are from the Cu NWs (Figure S1b) [33] within the Cu NWs–GO nanocomposite. The energy-dispersive X-ray spectroscopy (EDS) analysis in Figure 1e reveals the elemental composition of the prepared nanocomposite which shows the carbon, oxygen and copper elements, further proving the presence of GO and Cu, separately.

### 2.2. Voltammetric Behaviors of the Modified Electrode

**Figure 2:**
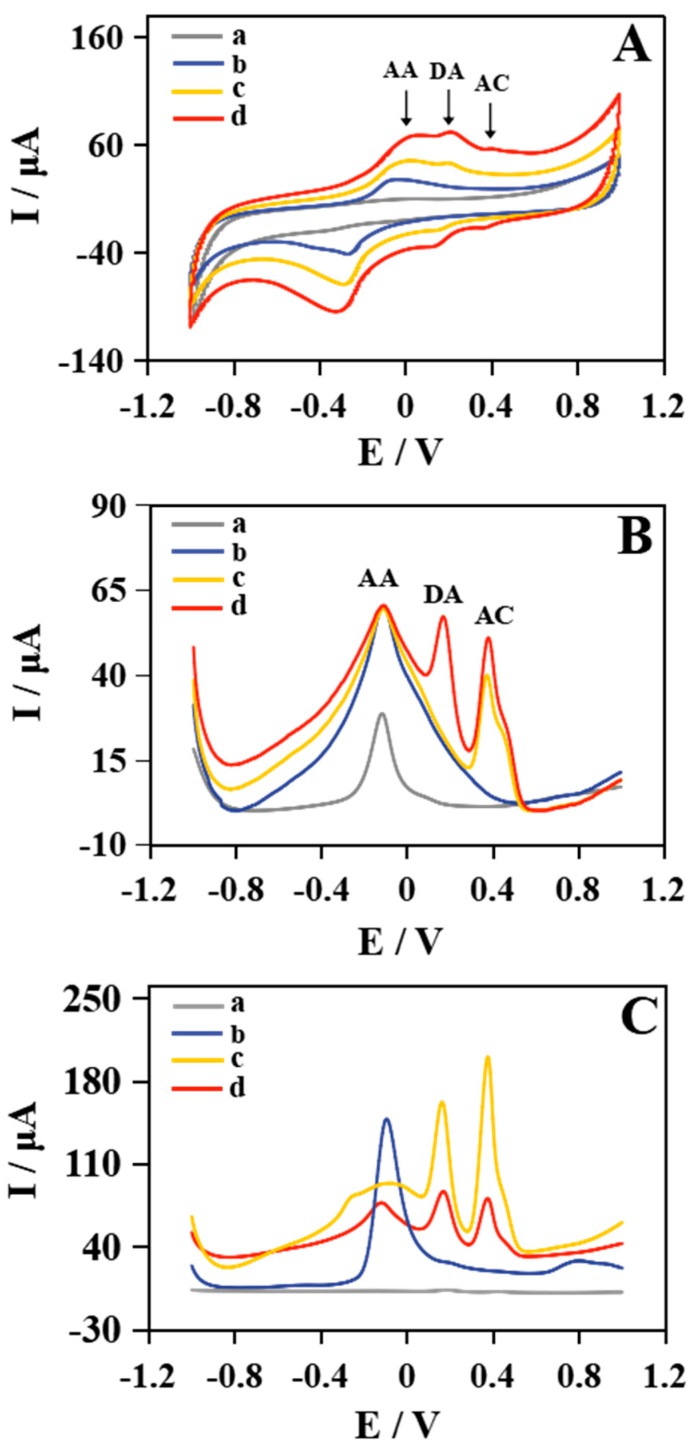
(**A**) Cyclic voltammetry (CV) of the Nafion/Cu NWs–GO/GCE with: (a) 0 µΜ ascorbic acid (AA), 0 µΜ dopamine (DA) and 0 µΜ acetaminophen (AC), (b) 10 µΜ AA, 0 µΜ DA and 0 µΜ AC, (c) 10 µΜ AA, 50 µΜ DA and 0 µΜ AC, and (d) 10 µΜ AA, 50 µΜ DA and 50 µΜ AC. (**B**) Differential pulse voltammetry (DPV) of the Nafion/Cu NWs–GO/GCE with: (a) 0 µΜ AA, 0 µΜ DA and 0 µΜ AC, (b) 10 µΜ AA, 0 µΜ DA and 0 µΜ AC, (c) 10 µΜ AA, 0 µΜ DA and 50 µΜ AC, and (d) 10 µΜ AA, 50 µΜ DA and 50 µΜ AC. (**C**) DPVs of (a) Nafion/GCE, (b) Nafion/Cu NWs/GCE, (c) Nafion/GO/GCE and (d) Nafion/Cu NWs–GO/GCE in 0.1 M phosphate-buffered saline (PBS) (pH 7.0) containing 10 µΜ AA, 50 µΜ DA, and 50 µΜ AC.

Cyclic voltammetry (CV) and DPV measurements were performed in 0.1 M phosphate-buffered saline (PBS) (pH 7.0) solution to evaluate the electrochemical property of Cu NWs–GO nanocomposite for simultaneous determination of AA, DA, and AC. Cyclic voltammetry was operated in the potential range from −1 V to +1 V with a scan rate of 100 mV s^−1^. As shown in the Figure 2A curve a, no response is observed in the absence of the three components on Nafion/Cu NWs–GO/GCE. A pair of redox peaks at about −0.05 V and −0.25 V is observed when adding AA, which is associated with the oxidation of hydroxyl groups to carbonyl groups of AA (curve b) [2,37,38,39]. A new couple of redox peaks at about +0.2 V and +0.15 V appeared with the continuing addition of DA, which corresponds to the redox reaction between DA and dopamine quinone (curve c) [5,40]. When AC coexisted with AA and DA in the PBS, a relatively low redox response of AC was acquired (curve d). The oxidation peak located at +0.4 V and the reduction peak at about +0.37 V of AC can be attributed to the redox reaction between AC and the quinonoid [41]. Cyclic voltammetry results confirm that the anodic oxidation of the three target molecules is a reversible process with three well-defined pair of redox peaks and no overlapped current signal on Cu NWs–GO nanocomposite modified GCE.

Differential pulse voltammetry was also utilized to analyze application of the three constituents’ simultaneous determination on account of the better resolution and higher sensitivity of this method. The DPV parameters that were carried out included a 50 mV amplitude and voltage range of −1 V to +1 V. Figure 2B depicts the DPV recordings of the separate signal of AA, DA and AC at Nafion/Cu NWs–GO/GCE. It can be seen in curve a that an obvious peak at −0.08 V appeared with the absence of the three components. This is caused by Cu NWs being easily oxidized to bivalent copper ions around 0 V during the positive scan [25,33]. Although the peak appeared here is consistent with the oxidation peak potential of AA, it does not affect the detection of AA as the twice response signal generated by adding AA (curve b). The result demonstrates the superiority of GO combined with Cu NWs, promoting the electro-oxidation of AA. This was further confirmed in a comparison test in Figure 2C. When AA was added to PBS, an anodic peak at −0.08 V was observed (curve b). After the addition of AC, one more peak at +0.38 V appeared (curve c). An anodic peak at +0.16 V was due to the DA oxidation (curve d). The three well-defined separated anodic peaks detected from DPV approximately corresponding to the CV results demonstrated the possibility for simultaneous testing of AA, DA, and AC on Nafion/Cu NWs–GO/GCE.

The voltammetric responses of different electrodes were compared as well to further prove the function of Nafion/Cu NWs–GO/GCE. Figure 2C displays the DPVs of the ternary mixture of 10 µΜ AA, 50 µΜ DA, and 50 µΜ AC in 0.1 M PBS (pH 7.0) at (a) Nafion/GCE, (b) Nafion/Cu NWs/GCE, (c) Nafion/GO/GCE, and (d) Nafion/Cu NWs–GO/GCE. No voltammetric peaks of AA, DA, and AC appeared on Nafion/GCE. For Nafion/Cu NWs/GCE, a strong voltammetric response appears around −0.08 V, consistent with the potential of AA oxidation peak, indicating the higher electrochemical activity of Cu NWs for AA. In addition, two sharp oxidation peaks occur at +0.16 V and +0.38 V, which are in accordance with those of DA and AC observed on Nafion/GO/GCE, respectively. However, a less distinct outline oxidation peak of AA around −0.08 V is indeterminacy of Nafion/GO/GCE. As expected, only the Nafion/Cu NWs–GO/GCE simultaneously shows three well-defined oxidation peaks at potentials of −0.08 V, +0.16 V, and +0.38 V. The above results show that Nafion/Cu NWs–GO/GCE shows a concurrently improved electrochemical activity for AA, DA, and AC. This may be due to the superiority of the prepared nanomaterials’ combined different functions within Cu NWs and GO. First, GO is rich in carboxylic acid, epoxy, phenol and hydroxyl groups on its basal planes and edges, giving GO the capability of adsorption and intercalation of electroactive species through hydrogen bonds, such as the proton-donating group of AA, DA, and AC [9]. Second, other than the GO serving as a site for a prominent electron acceptor, the modified electrochemical activity is attributed to the Cu NWs located on GO notably enlarging the specific electrochemical active areas and facilitating electron transfer rate, which can be conducive to electro-oxidation of AA, DA, and AC on the surface of a modified electrode. Third, the Cu NWs with superior electrochemical activity and outstanding conductivity capacitates this modified electrode to acquire a higher signal of electrochemical AA, as well as the effective separation of the three species. In consequence, the Cu NWs–GO nanocomposite-modified GCE exhibits a synergistic action for the simultaneous oxidation of AA, DA, and AC by combining and coordinating functions within Cu NWs and GO.

### 2.3. Optimization of Detection Conditions

**Figure 3:**
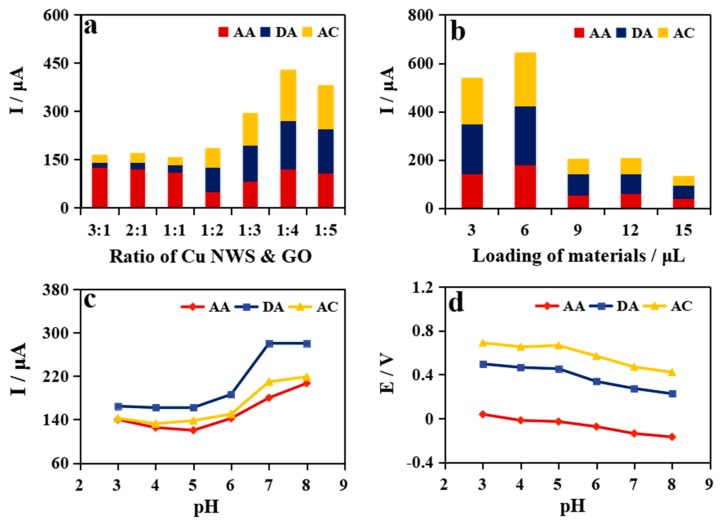
Optimum experiments of (**a**) the effect of the ratio of Cu NWs and GO, and (**b**) the loading of nanocomposite. Effect of pH on (**c**) peak current and (**d**) peak potential for the oxidation of 100 µΜ AA, 400 µΜ DA, 200 µΜ AC in 0.1 M PBS by DPV.

The simultaneous test signal of the Nafion/Cu NWs–GO/GCE towards AA, DA, and AC is affected by certain parameters, including the composition ratio of the Cu NWs and GO, the amount of nanocomposite loaded on the electrode, and the pH of the working electrolyte. Thus, experiments were carried out to estimate the impact of these parameters. In Figure 3a, the current response of AA is higher, with an increasing ratio of Cu NWs during the ratio of 1:1, 2:1, and 3:1 (*v*/*v*). This is further proved by the fact that Cu NWs have a good electrochemical oxidation effect with AA. Because of the larger ratio of GO, the current response of DA and AC increases progressively. As the results demonstrate, the ratio of Cu NWs to GO of 1:4 simultaneously reaches the maximum signal for the three analytes. With the optimal ratio, the loading amount of Cu NWs–GO nanocomposite was evaluated. Figure 3b shows that the current responses of the three compounds increase to a plateau at 6 μL Cu NWs–GO nanocomposite loading followed by a sharp decrease. Therefore, the ratio of Cu NWs to GO of 1:4 and 6 μL nanocomposites loading is selected for the following tests. Figure 3c and d present the pH influence on the simultaneous response to the three analytes in 0.1 M PBS containing 100 µΜ AA, 400 µΜ DA and 200 µΜ AC. As shown in Figure 3c, the peak currents of DA and AC increment with the increase of pH from 3.0 to 7.0 followed by a gradual saturation from 7.0 to 8.0. In the case of AA, the current signal initially decreases with pH ≤ 5 and then increases to reach a maximum of pH 8.0. Figure 3d shows that the three oxidation peak potentials from AA, DA and AC are shifted negatively by increasing pH, demonstrating that lower anodic potentials can distinguish the three molecules as well [9]. Therefore, pH 7.0, which is close to human physiological applications, was applied for subsequent work. In addition, the redox peak currents increases with varying scan rates from 40 mV s^−1^ to 240 mV s^−1^ (Figure S2). Good linear relationships between the peak currents and scan rates indicate that the electrooxidation of AA, DA, and AC on Cu NWs–GO nanocomposite modified GCE is a surface-controlled process.

### 2.4. Simultaneous Detection of Ascorbic Acid (AA), Dopamine (DA), and Acetaminophen (AC) in Nafion/Cu NWs–GO/GCE

Under optimal conditions, the individual detections of AA, DA, and AC were first performed in 0.1 M PBS (pH 7.0) by DPV (Figure S3a–c). Their approximate peak potentials are −0.1 V, +0.16 V, and +0.38 V, respectively. The peak current responses of the three compounds increase with the increment of their concentrations. Figure S3d–f show the calibration plots for the DPV responses of AA, DA, and AC in the ranges from 1 μM to 86 μM (I(AA) = 1.0952 × C(AA) + 74.559, R^2^ = 0.9674), 1 μM to 120 μM (I(DA) = 0.8814 × C(DA) + 3.5879, R^2^ = 0.9794), and 1 μM to 90 μM (I(AC) = 1.9479 × C(AC) + 11.304, R^2^ = 0.9883), respectively. The detection limits are calculated to be 0.44 μM, 0.07 μM and 0.19 μM (S/N = 3), respectively. These results indicate that the excellent electrochemical activity of Cu NWs–GO nanocomposite provides a possible foundation for individual detection of AA, DA, and AC.

Next, the concentration of one target analyte that was only changed in the mixture was investigated on the basis that the concentrations of the other two analytes were kept constant. As shown in Figure S4a, three peaks located at −0.1 V, +0.16 V and +0.38 V, corresponding to the oxidation of AA, DA, and AC, are well separated in Nafion/Cu NWs–GO/GCE. The peak current of AA oxidation increases linearly in the range of 1–60 μM with the detection limit of 0.11 μM in the presence of 10 μM DA and 10 μM AC, which does not affect the peak potentials of DA and AC. The linear function of AA is I(AA) = 1.7439 × C(AA) + 104.79 (R^2^ = 0.9604) (Figure S4d). Figure S4b describes the representative oxidation current response variation with different DA concentrations between 1 μM to 160 μM and constant concentrations of AA (10 μM) and AC (10 μM). Figure S4e exhibits two linear regions from 10 μM to 80 μM (I(DA)1 = 2.1328 × C(DA) + 31.616, R^2^ = 0.998) and from 80 μM to 160 μM (I(DA)2 = 0.6041 × C(DA) + 154.13, R^2^ = 0.9851), respectively. And the detection limit is 0.39 μM. Similarly, the current signal of AC increases with the increment of its corresponding concentrations between 1μM to 110 μM under constant concentrations of AA (10 μM) and DA (10 μM) in Figure S4c,f. The corresponding linear equation is I(AC) = 0.6008 × C(AC) + 12.387 (R^2^ = 0.9917), with a detection limit of 0.07 μM. These experimental results illustrate a possible synergy between Cu NWs and GO to simultaneously detect the three biomolecules.

**Figure 4:**
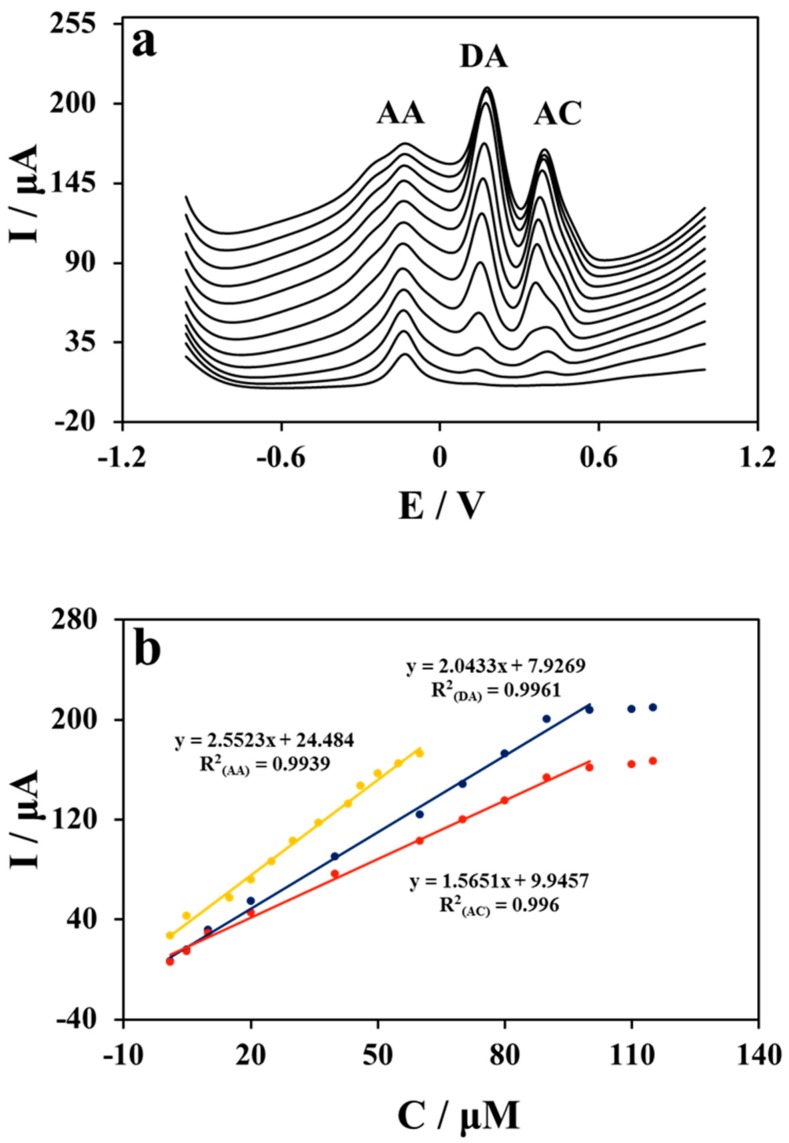
(**a**) DPV profiles at Nafion/Cu NWs–GO/GCE in 0.1 M PBS (pH 7.0) containing different concentrations of AA, DA, and AC. From bottom to up the concentrations: 1, 5, 15, 20, 25, 30, 36, 43, 46, 50, 55, 60 μΜ for AA; 1, 5, 10, 20, 40, 60, 70, 80, 90, 100, 110, 115 μΜ for DA; 1, 5, 10, 20, 40, 60, 70, 80, 90, 100, 110, 115 μΜ for AC. (**b**) Plots of the oxidation peak currents of AA, DA and AC vs. concentrations.

As a consequence, the simultaneous testing of AA, DA, and AC was implemented by voltammetric methods by synchronously and orderly changing the concentrations from low to high of the three biomolecules. It is obvious from Figure 4a that the three oxidation peaks are non-overlapping and well separated, and the peak currents increase with the successive addition of AA, DA, and AC in greater concentrations. The corresponding calibration plots are presented in Figure 4b, which are linear in the concentration ranges of 1–60 μM, 1–100 μM, and 1–100 μM for AA, DA, and AC with detection limits of 0.05μM, 0.41 μM and 0.04 μM (S/N = 3), respectively. The corresponding fitting equations of AA, DA, and AC are calculated as I(AA) = 2.5523 × C(AA) + 24.484 (R^2^ = 0.9939), I(DA) = 2.0433 × C(DA) + 7.9269 (R^2^ = 0.9961), and I(AC) = 1.5651 × C(AC) + 7.6101 (R^2^ = 0.996), respectively. The results demonstrate that the simultaneous discrimination of the three biomolecules in Cu NWs–GO composite is practicable in a mixture solution via DPV, and with wider linear ranges and/or lower detection limits than previously reported other nanomaterial modified electrodes (listed in Table 1), demonstrating that simultaneous testing of the three biomolecules can be performed successfully on Nafion/Cu NWs–GO/GCE with high sensitivity.

### 2.5. Interference, Reproducibility and Stability

Glucose, chloride ion, sodium ion, and potassium ion, which exist abundantly in human blood, are commonly considered to impose a major issue on simultaneous detection of AA, DA, and AC [44,47,48,49]. In addition, uric acid, an endogenous reducing compound, is in co-existence with AA and DA in physiological conditions, which is considered to be the major interfering specie for the simultaneous detected sensors, especially including DA sensing, due to their closer oxidation potential [47,50]. Therefore, the selectivity of the as-prepared Nafion/Cu NWs–GO/GCE against the four interfering substances was examined. The normal physiological concentration of glucose is about 3–8 mM in human blood and the normal level of AA and UA are about 0.1 mM [48]. The presence of the optimum level of DA is 26–40 nM under the physiological condition [51]. Referring to the similar studies measured the concentrations of inorganic ions [22,46], a challenging selectivity study was conducted using high concentration of glucose (10 mM), UA (20 μM), KCl (1 mM), and NaCl (1 mM) compared to AA (1 µΜ), DA (1 µΜ), and AC (1 µΜ). The selectivity experiment was evaluated by simultaneous addition of the four interference compounds to 0.1 M PBS in the presence of AA, DA, and AC through DPV. It is found in Figure S5 that 10,000-fold glucose, 20-fold UA, and 1000-fold Na^+^, K^+^, and Cl^−^ do not significantly interfere with the determination of AA, DA, and AC. The results demonstrate that the simultaneous determination of AA, DA, and AC were reliable via DPV, and with better selectivity than previously reported the other nanomaterials modified electrodes (listed in Table S1) [22,46], indicating that simultaneous testing of the three biomolecules can be successfully performed on Nafion/Cu NWs–GO/GCE.

**Figure 5:**
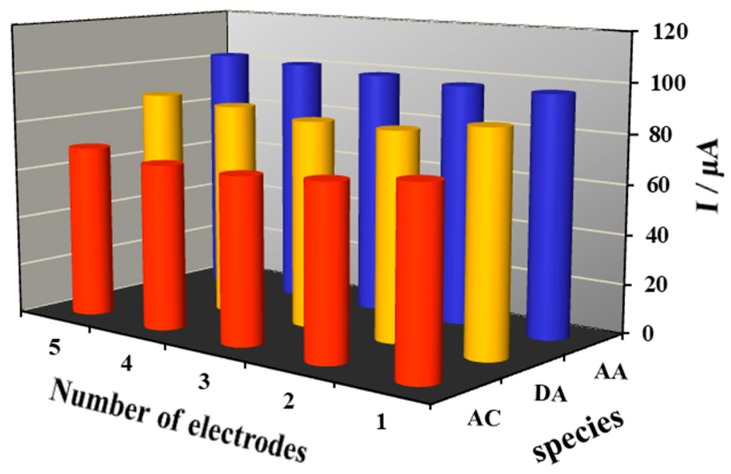
Reproducibility studies of a series of 5 electrodes with 30 µΜ AA, 45 µΜ DA, 45 µΜ AC using DPV in 0.1 M PBS (pH 7.0) solution.

Reproducibility was examined by analysis in a mixture of 30 µΜ AA, 45 µΜ DA, and 45 µΜ AC in 0.1 M PBS (pH 7.0) solution, applying five electrodes under the same modified conditions (Figure 5). The low relative standard deviations (RSD) of 2.4%, 2% and 2.5% for AA, DA, and AC, respectively, demonstrate good reproducibility of the prepared electrodes. Furthermore, the stability of the prepared working electrode was investigated for 12 days’ storage in ultrapure water at 4 °C. Before and after storage, the response signals of AA, DA, and AC decreased by 5.4%, 2.1%, and 4.1% of their original current, respectively, demonstrating the good stability of the developed method.

### 2.6. Detection Application in Human Serum Sample

Nafion/Cu NWs–GO/GCE was employed to simultaneously determine AA, DA, and AC in human serum samples with the standard addition method. The human serum samples were pretreated by centrifugation at 6000 rpm for 5 min, and the collected supernatants were diluted 100 times with 0.1 M PBS (pH 7.0). The measured concentrations were calculated by the calibration plots shown in Figure 4b. As is seen in Table 2, the appropriate recoveries in the range of 94.6–101.7%, and small RSD ranges between 0.6% and 3.3%, indicate the reliability of Cu NWs–GO nanocomposite in the application of simultaneous testing of AA, DA, and AC in human serum samples.

## 3. Experiment

### 3.1. Reagents and Materials

Uric acid, ascorbic acid, dopamine hydrochloride, copper(II) nitrate hemipentahydrate (Cu (NO_3_)_2_•2.5H_2_O), ethylenediamine (>99.9%), Nafion 117 solution, hydrazine, sodium hydroxide and glucose were purchased from Sigma-Aldrich (St. Louis, MO, USA). Acetaminophen was purchased from Alfa Aesar (Heysham, England). Graphene oxide was obtained from XFNANO Nanotechnology Co., Ltd. (Nanjing, Jiangsu, China), which is also the manufacturer of GO. Disodium hydrogen phosphate dodecahydrate, sodium chloride, potassium chloride, ethanol, acetone and sodium dihydrogen phosphate dehydrate were purchased from Chongqing Chuandong Chemical Group Co., Ltd. (Chongqing, China). Human serum samples were used as received from the First Affiliated Hospital of Chongqing Medical University. Ultrapure water (18.2 MΩ cm) was applied for preparing all solutions. 

### 3.2. Characterization and Electrochemical Measurements

The microstructure characterizations of the prepared materials were accomplished on a SU8010 scanning electron microscope (SEM, Hitachi, Japan) which was equipped with X-Max^N^ energy-dispersive X-ray spectroscopy (EDS, Oxford Instruments, UK). The crystal structures of the samples were carried out on an Empyrean X-ray Diffraction (XRD) System (Empyrean, PANalytical, Almelo, The Netherlands). All electrochemical tests were performed on a CHI 660D electrochemical workstation (Shanghai CH Instruments Co., Shanghai, China) and AUTOLAB PGSTAT302N (Metrohm Autolab, Herisau, Switzerland). A three-electrode system was commonly utilized with a 3-mm-diameter glassy carbon electrode as the working electrode, Ag/AgCl (3 M KCl) electrode as the reference electrode, and Pt wire as the counter electrode.

### 3.3. Preparation of Nanocomposite Modified Electrode 

The commercial GO was dispersed in ultrapure water with sonication for 4 h to obtain homogenous dispersion. Copper NWs were prepared based on previous reports [25,33]. A mixed solution of 20 mL NaOH solution (15 M), 1 mL Cu(NO_3_)_2_ •2.5H_2_O (0.1 M), 0.16 mL ethylenediamine and 25 µL hydrazine (35 wt%) was reacted at 60 °C for 2 h. The Cu NWs end product was adequately washed with ethanol and ultrapure water, separately, and then dispersed in ethanol for further use. Next, a given volume ratio of Cu NWs (2 mg/mL) and GO (2 mg/mL) was mixed and sonicated for 5 h at room temperature to obtain uniform Cu NWs–GO nanocomposite suspension. An appropriate amount of Cu NWs–GO nanocomposite suspension was directly dropped on the surface of bare GCEs which were cleaned carefully with conventional polished processes with alumina slurries. Then, 20 μL of Nafion ethanol solution (0.1 wt %) was cast on the modified layer for the purpose of entrapment and long-term stability. After drying at room temperature, the modified electrode was readily prepared and denoted as Nafion/Cu NWs–GO/GCE. The control electrodes were also prepared in a similar way.

## 4. Conclusions

Copper NWs modified with GO nanosheets for simultaneous electrochemical analysis of AA, DA, and AC were investigated. The manufacture of Cu NWs–GO nanocomposite went through a simple sonication technique. It was successfully characterized by SEM, XRD, and EDS. The Cu NWs–GO nanocomposite with a Nafion-modified glassy carbon electrode exhibits three well-defined peaks from AA, DA, and AC. The electrochemical property studies verify the synergistic effect of Cu NWs and GO. The specific surface, electrochemical oxidation performance and conductivity of the nanocomposite are improved when compared to each individual component. The current responses of the prepared electrode for AA, DA, and AC are simultaneously changed regularly with a wide linear range and a low detection limit via DPV. The good reproducibility, selectivity, and stability demonstrate its outstanding applicability to simultaneous testing of AA, DA, and AC. The method successfully applied in human serum samples reveals that the Cu NWs–GO nanocomposite is a promising candidate for AA, DA and AC simultaneous detection.

## Figures and Tables

**Table 1:** Comparison of different electrodes for the responses of AA, DA, and AC at room temperature.

Electrodes	Methods	Linear Ranges (μM)			Detection Limit (μM)			Ref.
		AA	DA	AC	AA	DA	AC	
GO-XDA-Mn_2_O_3_/GCE	Chronoamperometric	10–8000	-	1–1000	0.6	-	0.056	[29]
MWCNT/GO/AuNR/GCE	DPV	1–8000	-	-	0.087	-	-	[24]
GO-ZIF67/GCE	DPV	-	0.2–80	-	-	0.05	-	[28]
p-GLY/GO/GCE	DPV	-	0.2–62	-	-	0.011	-	[31]
AgNPs/P(Arg)-GO/GCE	DPV	4–2400 (only change C_AA_)	0.05–50 (only change C_DA_)	-	0.984	0.01	-	[30]
Cu_2_O/GR/GCE	DPV	-	-	0.02-1.3	-	-	0.0067	[42]
CuZEA/RGO/GCE	DPV	20–200 (only change C_AA_)	0.1–19 (only change C_DA_)	-	11	0.041	-	[43]
Zn-NiAl LDH/rGO/GCE	DPV	0.5–11	0.001–1	-	0.0135	0.0001	-	[44]
3D-MoS_2_/rGO/Au/GCE	DPV	2–5400	0.3–198.3	-	1.46	0.15	-	[23]
RGO-CdSe QD/GCE	DPV	390–1000 (only change C_AA_)	4.9–74 (only change C_DA_)	-	66	0.11	-	[45]
Fc-S-Au/C NC/graphene/GCE	DPV	8–400	0.2–2.5	0.5–46	1.0	0.05	0.1	[17]
Cu^2+^@PDA-MWCNTs/GCE	DPV	5–175	4–125	5–75	0.82	0.45	0.87	[46]
Fe_3_O_4_@Au-S-Fc/GS-chitosan/GCE	DPV	6–350	0.5–50	0.4–32	1.0	0.1	0.05	[16]
PSNSB/CPE	DPV	2.5–1050	0.05–120	0.033–158	0.02	0.002	0.005	[22]
Au/ZnO/N-doped graphene/GCE	DPV	30–13,000 (only change C_AA_)	2–180 (only change C_DA_)	5–3100 (only change C_AC_)	5.0	0.4	0.8	[1]
Cu NWs/GO/GCE	DPV	1–60	1–100	1–100	0.05	0.41	0.04	this work

**Table 2:** The simultaneous determination of AA, DA, and AC in human serum samples.

Samples	Added (μM)			Found (μM)			Recoveries			Relative Standard Deviation (RSD) (%) (n = 5)		
	AA	DA	AC	AA	DA	AC	AA	DA	AC	AA	DA	AC
1	50.0	68.0	70.0	49.1	66.9	71.2	98.2	98.4	101.7	3.3	1.9	2.1
2	52.0	72.0	75.0	50.3	68.0	71.0	96.8	94.6	94.7	1.6	0.8	1.4
3	54.0	74.0	78.0	51.8	70.1	73.8	96.0	94.8	94.6	1.0	0.6	0.8

## Supplementary Materials

The following are available online. Figure S1. The XRD patterns of (a) GO and (b) Cu NWs; Figure S2. (a) Cyclic voltammograms of the Nafion/Cu NWs-GO/GCE in 0.1 M PBS (pH 7.0) containing 20 µΜ AA, 50 µΜ DA, and 50 µΜ AC at different scan rates (40–240 mV•s-1) and the corresponding plots of current vs. scan rate of (b) AA, (c) DA, and (d) AC; Figure S3. DPVs of Nafion/Cu NWs-GO/GCE in 0.1 M PBS (pH 7.0) containing (a) AA: 1, 5, 10, 15, 20, 35, 45, 55, 60, 66, 72, 76, 80, 86 μΜ; (b) DA: 1, 5, 10, 30, 40, 50, 60, 80, 100, 120 μΜ; and (c) AC: 1, 5, 10, 20, 40, 50, 80, 90, 100, 110 μΜ. The corresponding calibration plots of (d) AA, (e) DA, and (f) AC, respectively; Figure S4. DPVs at Nafion/Cu NWs-GO/GCE in 0.1 M PBS (pH 7.0); Figure S5. Interference test of Nafion/Cu NWs-GO/GCE upon the addition of different substances, DPVs with (a) 1 µΜ AA, 1 µΜ DA, 1 µΜ AC and with (b) addition of 10 mM glucose, 20 μM UA, 1 mM NaCl and 1 mM KCl. Table S1. Comparison of different electrodes on selectivity study.
