# Supplementary material for: Copper Nanowires Modified with Graphene Oxide Nanosheets for Simultaneous Voltammetric Determination of Ascorbic Acid, Dopamine and Acetaminophen
Source: Molecules. 2019 Jun 24;24(12):2320. doi: 10.3390/molecules24122320 (PMC6631772; doi:10.3390/molecules24122320)
Supplement: Supplementary file 1 [file molecules-24-02320-s001.pdf]

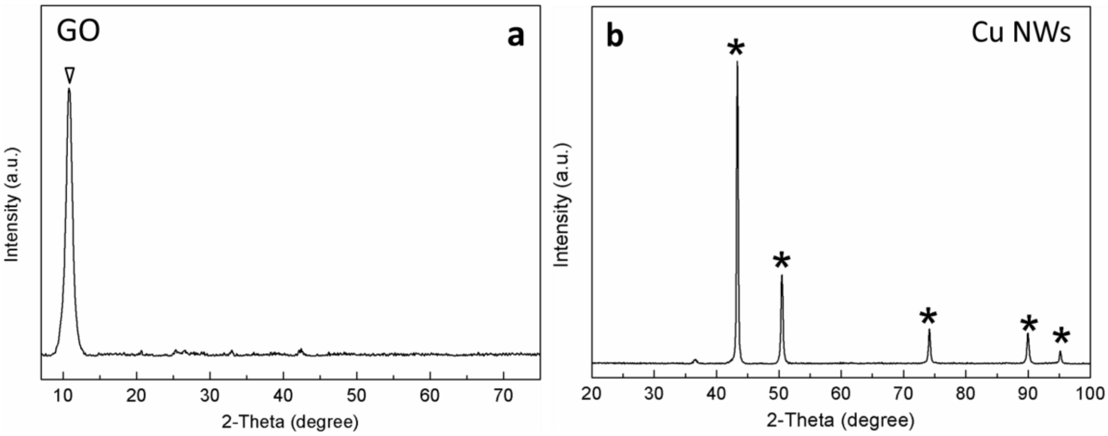

Figure S1. The XRD patterns of (a) GO and (b) Cu NWs.

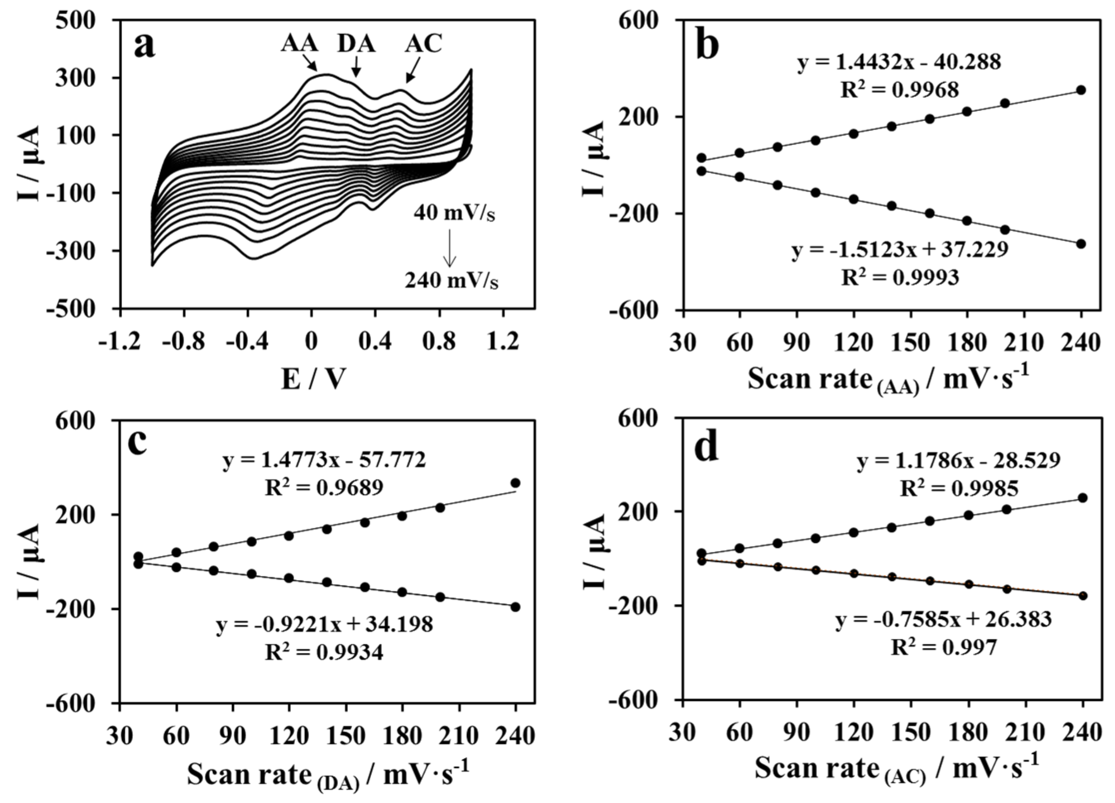

Figure S2. (a) Cyclic voltammograms of the Nafion/Cu NWs-GO/GCE in 0.1M PBS (pH 7.0) containing 20 μM AA, 50 μM DA, and 50 μM AC at different scan rates (40-240 mV•s^{-1}) and the corresponding plots of current vs. scan rate of (b) AA, (c) DA, and (d) AC.

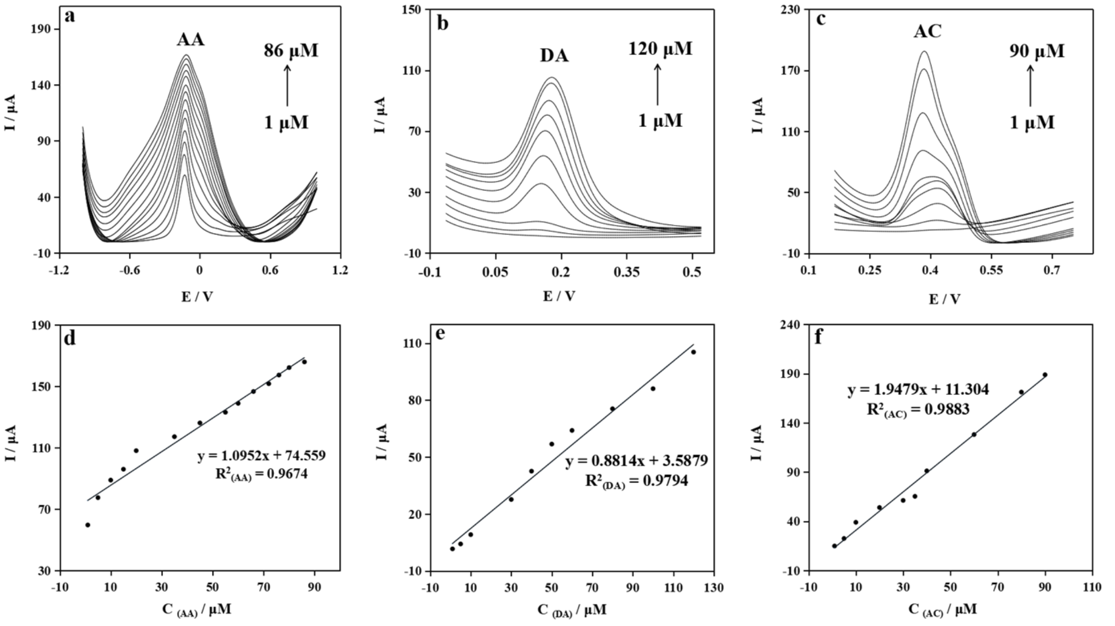

Figure S3. DPVs of Nafion/Cu NWs-GO/GCE in 0.1M PBS (pH 7.0) containing (a) AA: 1, 5, 10, 15, 20, 35, 45, 55, 60, 66, 72, 76, 80, 86 μM; (b) DA: 1, 5, 10, 30, 40, 50, 60, 80, 100, 120 μM; and (c) AC: 1, 5, 10, 20, 40, 50, 80, 90, 100, 110 μM. The corresponding calibration plots of (d) AA, (e) DA, and (f) AC, respectively.

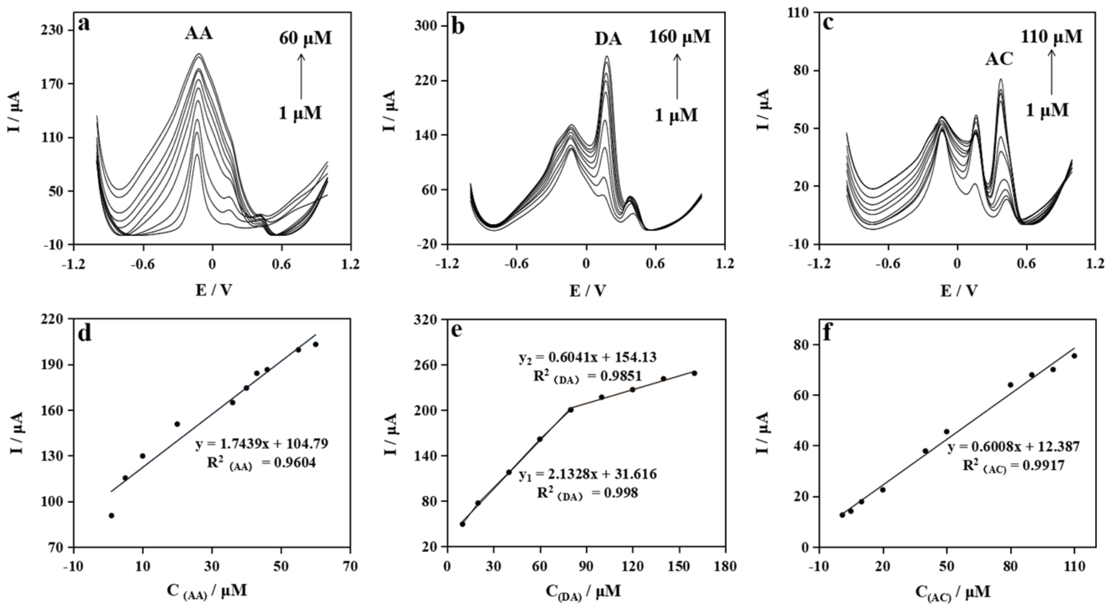

Figure S4. DPVs at Nafion/Cu NWs-GO/GCE in 0.1 M PBS (pH 7.0) (a) containing 10 µM DA, 10 µM AC and different concentrations of AA: 1, 5, 10, 20, 36, 40, 43, 46, 55, 60 µM; (b) containing 0.01 µM AA, 10 µM AC and different concentrations of DA : 10, 20, 40, 60, 80, 100, 120, 140, 160 µM; (c) containing 10 µM AA, 10 µM DA and different concentrations of AC : 1, 5, 10, 20, 40, 50, 80, 90, 100, 110 µM. Plots of the anodic peak currents vs. (d) AA, (e) DA and (f) AC concentrations, respectively.

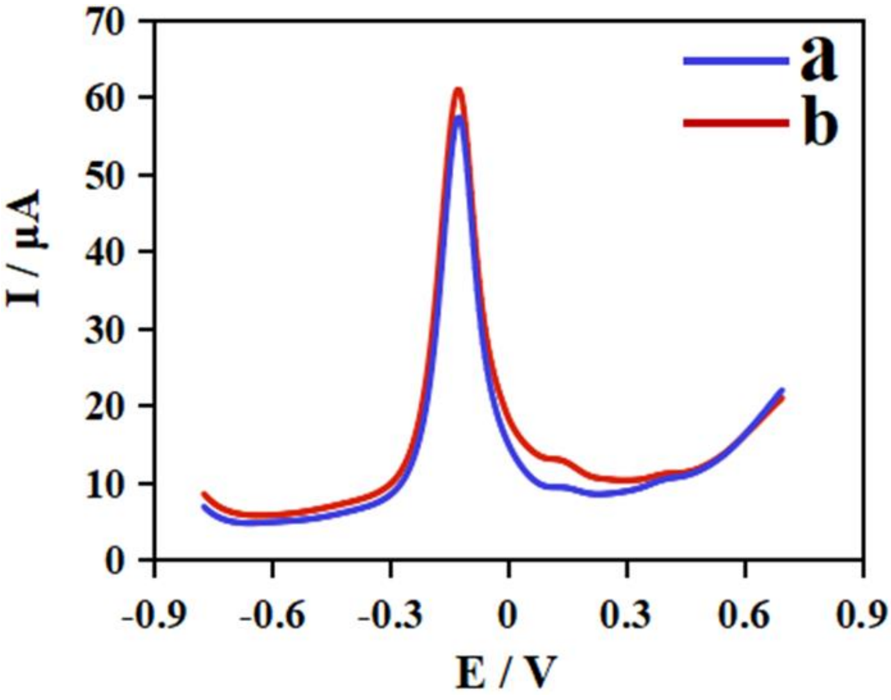

Figure S5. Interference test of Nafion/Cu NWs-GO/GCE upon the addition of different substances, DPVs with (a) 1 μM AA, 1 μM DA, 1 μM AC and with (b) addition of 10 mM glucose, 20 μM UA, 1 mM NaCl and 1 mM KCl.

Table S1. Comparison of different electrodes on selectivity study.

Foreign species	Ref. 22			Ref.46			This work		
	AA (20 μM)	DA (100 μM)	AC (50 μM)	AA (25 μM)	DA (25 μM)	AC (25 μM)	AA (1 μM)	DA (1 μM)	AC (1 μM)
Glucose	300 μM	300 μM	400 μM	80 μM	80 μM	100 μM	10000 μM	10000 μM	10000 μM
Uric acid	-	-	-	20 μM	15 μM	8 μM	20 μM	20 μM	20 μM
Na^+	400 μM	1000 μM	350 μM	1000 μM	1000 μM	1000 μM	1000 μM	1000 μM	1000 μM
K^+	400 μM	1000 μM	350 μM	1000 μM	1000 μM	1000 μM	1000 μM	1000 μM	1000 μM
Cl^-	300 μM	1000 μM	400 μM	1000 μM	1000 μM	1000 μM	1000 μM	1000 μM	1000 μM
